# Applications Used to Increase the Accuracy Rate of Transthoracic Lung Biopsies Performed in Centers for Molecular Biological Diagnosis and Targeted Therapy for Oncology

**DOI:** 10.7759/cureus.54049

**Published:** 2024-02-12

**Authors:** Murat Asik, Ayten Guner Akbiyik

**Affiliations:** 1 Department of Radiology, Istanbul Medeniyet University, Istanbul, TUR; 2 Radiology, Prof. Dr. Suleyman Yalcin City Hospital, Istanbul, TUR; 3 Thoracic Surgery, Prof. Dr. Suleyman Yalcin City Hospital, Istanbul, TUR

**Keywords:** trans thoracic biopsy, minimally invasive interventional radiology, radiological imaging techniques, core needle biopsy, lung cancer (lc)

## Abstract

Purpose

This study aims to assess the significance of imaging techniques and needle thickness employed in transthoracic core needle biopsy for determining the cancer type and subtypes, ultimately guiding the treatment of lung cancer.

Material and methods

Between 2018 and 2023, a cohort of 350 patients (69.7% male, 30.3% female) underwent CT-guided lung biopsy, predominantly utilizing core biopsies. Fine needle aspiration biopsies employed 18 or 20 G Chiba needles, while core needle biopsies utilized 16 or 18-gauge coaxial system semi-automatic needles. The preferred needle and biopsy sample size were 16 G in thickness and 2 cm in length. Pre-procedure positron emission tomography-computed tomography (PET-CT) images aided in identifying the most homogenous lesion with the highest SUV max value, guiding biopsy sample extraction. Post-procedure control CT evaluated complications according to the Society of Interventional Radiology (SIR) reporting standard.

Results

The average age of biopsied patients was 65.48 +/- 12.32 SD (range: 18-90). Tru-cut biopsy was predominant (69.7%), utilizing a larger number of 16G needles. Pathological diagnoses were mostly malignant (76.6%), with lesion sizes averaging 35.98 +/- 17.90 SD (range: 5-105 mm) and distances to pleura averaging 13.48 +/- 13.54 SD (range: 0-86 mm). Malignancy prevalence was higher in males (56.8%), tru-cut biopsies (72.7%), 16G needles used for tru-cut (47.7%), and PET-CT evaluation (59.1%). Complications were identified in 22% of cases, with distance to pleura significantly associated (p < 0.001). No significant differences in complication risk were observed between FNAB and tru-cut and between needle gauges (20 G-18 G and 16 G) (p: 0.734, p: 0.638, respectively).

Conclusion

The study underscores the paramount importance of biopsy sample size in diagnosing lung cancers and determining targeted therapy. Optimal biopsy localization, informed by pre-procedure imaging techniques, is crucial. Hence, the recommendation is to utilize the thickest needles and largest samples for lung biopsies.

## Introduction

The most commonly seen and leading cause of death due to cancer worldwide is lung cancer [1]. Medical treatment is used for cancers other than small cell carcinoma and non-small cell carcinoma, which only has 30% of surgical treatment. Recently, with the developments in target therapy in oncology centers, systemic chemotherapy has taken a back seat, and target therapies specific to cancer type and even the individual person have been given preference [2]. However, for these treatments, an adequate tissue sample should be taken to determine the type/subtype of cancer. Once an adequate tissue sample is taken, the molecular fingerprint of somatic mutation can be studied in cancer cells for this purpose. To diagnose lung lesions, transthoracic core needle biopsy (TCNB) and fine needle aspiration biopsy (FNAB) are performed. TCNB has become more commonly used to diagnose lung lesions due to its high diagnostic value and ability to obtain sufficient tissue to determine the type of cancer [3]. The accuracy rate of TCNB is stated to be higher than that of FNAB, with accuracy rates ranging from 50% to 96% [4,5]. The choice between FNAB or TCNB shall be based on the radiologic characteristics of the lesion (size, location, neighborhood, density), the acceptable rate of biopsy complications that may occur, and the purpose of the procedure [6,7]. However, since most patients are not treated surgically and nowadays, personalized individual treatments are taking precedence, molecular tissue diagnosis and biomarker studies are more sought after by oncology centers and interventional radiologists than just diagnostics. For this reason, CT-guided needle biopsies are preferred as first-line approaches for lung biopsies, regardless of the lesion's size [8,9]. Additionally, instead of multiple needle biopsy entries to obtain more samples, thicker needles and longer specimens are attempted to be taken for a more reliable sample. The aim of this study is to identify the available techniques to obtain more tissue samples for molecular biological studies prior to target treatment planning in interventional radiology units operating at oncology centers for primary malignantly suspected lesions in the lung.

## Materials and methods

Patient population

Between 2018 and 2023, 350 patients with suspected malignancy who underwent CT-guided lung biopsy were included in the study (male: 244 (69,7%), female: 106 (30,3%)). Pre- and post-biopsy imaging findings and pathological diagnoses were reviewed retrospectively. All patients were provided with comprehensive information regarding the procedure, and written consent was obtained from each of them, indicating their understanding and agreement to undergo the aforementioned medical intervention. Ethics committee approval (ethical no:2019/0291) was obtained and the study was conducted in accordance with the principles of the Declaration of Helsinki. Biopsy decisions for the patients were made by a multidisciplinary council consisting of pulmonology, thoracic surgery, medical oncology, and diagnostic and interventional radiology. In cases of patients with a lung tumor, the initial step was to perform bronchoscopy in order to localize the lesion. If the lesion could not be diagnosed through bronchoscopy or was deemed unsuitable for the procedure, a percutaneous transthoracic biopsy was carried out to obtain tissue samples. Most of the patients had thorax CTs together with positron emission tomography-computed tomography (PET-CT) scans prior to biopsy (Table 1). Patients without any coagulation disorder in coagulation tests (Prothrombin Time (PT)/Activated Partial Thromboplastin Time (aPTT)/International Normalized Ratio (INR)) were accepted. Adult patients over 18 years old, with platelet count above 50.000/mm^3^, INR ≤1.5, and those who could lie in a supine/prone/decubitus position during the biopsy on the CT table under local anesthesia were included in the study. The exclusion criteria for this study included patients for whom imaging findings and pathology results were unavailable, lesions with a diameter smaller than 5 mm, patients with bleeding and coagulation disorders, individuals who experienced anxiety preventing them from lying down, and those with whom effective communication was not possible.

**Table 1:** Demographic data and characteristics of pulmonary lesions N:  number of data; SD: standard deviation; FNAB: fine needle aspiration biopsy; PET-CT: positron emission tomography–computed tomography, SUV: standardized uptake value

Parameter	Value N (%)
Total number of patients	350
Sex	
Male	244 (69.7%)
Female	106 (30.3%)
Age (mean ± SD)	65.48 ± 12.32 years
Biopsy type	
Tru-cut	244 (69.7%)
FNAB	106 (30.3%)
Needle gauge	
16 G	173 (49.4%)
18 G	86 (24.6%)
20 G	91 (26%)
Pathological diagnosis	
Malignant	268 (76.6%)
Benign	44 (12.6%)
Unknown	38 (10.9%)
Lesion size (mean ± SD)	35.98 ± 17.90 mm
Distance from pleura (mean ± SD)	13.48 ± 13.54 mm
PET-CT availability	
Yes	240 (68%)
No	110 (32%)
SUVmax (mean ± SD)	6.65 ± 6.34

Procedure

Before the biopsy procedure, patients underwent assessments of respiratory function, coagulation status, and comorbidities. Administration of antiplatelet medications and oral anticoagulants was halted five days before, and low molecular weight heparins were discontinued 24 hours prior to the procedure. For patients on a new oral anticoagulant regimen, the previous dose was stopped a day prior, and the initial postoperative dose was administered in the evening. The prebiopsy evaluation aimed for platelet counts of 50,000/mm^3^ and accepted INR ≤ 1.5.

The largest dimension of the lesion to be biopsied was determined, considering its location in the pulmonary parenchyma, proximity to the main bronchi and vascular structures, and distance from the pleura. The decision between Fine Needle Aspiration Biopsy (FNAB) or tru-cut biopsy was made in conjunction with a 128-detector CT (OPTIMA 660 General Electric Medical Systems, Milwaukee, WI, USA). Imaging before the biopsy aimed to avoid lungs, fissures, large vascular structures, and bones to identify an appropriate biopsy location.

After reviewing previous scans, metallic markers were placed on the skin by estimating the lesion's imprint. The panoramic acquisition (scout) image aligned the lesion and markers. The first scan was 5 mm thick, and subsequent scans were performed with a section thickness range of 0.625-2.5 mm, considering the lesion's size, location, and surrounding area. Low-dose scanning (120 kVp, 30 mAs) was considered. Needle entry localization was determined by gantry laser light and manually placed radio-opaque metallic grids.

After the first scan, the distance from the skin to the pleura and the pleura to the lesion was measured on the computer, and the needle length was determined. The position and entry point for the biopsy were determined, and the skin was disinfected with povidone-iodine before applying 1% lidocaine for local anesthesia from the skin to the pleural surface.

For patients undergoing Core Needle Biopsy (CNB), a small precise cut was made using a scalpel at the designated entry point, and the core biopsy needle was carefully positioned and secured in the skin. The needle's direction toward the lesion was adjusted with retraction and advancement until it reached the lesion. After sampling from the lesion, the process was repeated or concluded based on the integrity, thickness, and length of the sample taken in formalin.

If FNAB was performed, the sample smears were evenly spread on a slide using 18 or 20G Chiba needles (Geotek, Ankara, Turkey). Half of the samples were air-dried, while the other half was fixed in 90% alcohol and sent for pathological examination. If CNB needed to be performed, 16 or 18-gauge coaxial system semi-automatic needles (Geotek, Ankara, Turkey) were used. The body of these needles was made of plastic and was small and very light, making them easily secured in place without moving from their position after the initial insertion from the skin. More often, the first preferred needle of 16 G thickness and 2 cm length was chosen to obtain more tissue samples during initial entry. The multidisciplinary council decided to prioritize core biopsy, using the largest available cutting needle for biopsy techniques. According to this decision, biopsies were preferably performed using a 16G needle; if not, an 18G needle for core biopsy.

Under optimal conditions, an attempt was made to obtain a sample that was at least 15 mm long, instead of 10 mm, from the entry point of the lesion to the longest dimension of the lesion. In cases where core biopsy was not possible, FNAB was performed with an 18G needle instead of a 20G. Low-weight semi-automated needles were used for core biopsies. Needles of length 10 cm or 15 cm were used according to the distance of the lesion to the visceral pleura. The decision to place the patient in a position (anterior-posterior-decubitus) according to the lesion's location was made.

Then, PET-CT images and reports were examined. Biopsy was taken from the area with the highest SUV max value, and the lesion's most homogeneous FDG uptake was reported in the report. Multiple samples were taken if the patients tolerated it and there were no complications. After the biopsy, a control CT was taken to see if there were any complications. Complications were evaluated and graded according to the Society of Interventional Radiology (SIR) reporting standard [10]. Patients with complications underwent clinical observation. After two hours, Posterior Anterior (PA) chest radiographs were obtained and evaluated. In all patients with complications, the complications regressed spontaneously without the need for thoracic tube drainage and additional interventional treatment.

Statistical evaluation

Statistical analysis was conducted using SPSS software, version 26.0 (IBM Corp., Armonk, NY). The normal distribution of variables was examined using the Shapiro-Wilk test. Descriptive statistics, represented as mean ± standard deviation for continuous variables and percentages for categorical variables were employed. Group comparisons utilized t-tests, Chi-square tests, and Mann-Whitney U-tests. In cases with more than two groups, the ANOVA test was applied. Logistic regression analysis was employed to assess the synergistic effects of variables positively influencing diagnostic biopsy and those posing a risk for complications. A significance level of P < 0.05 was considered statistically significant.

## Results

A total of 350 patients who underwent lung biopsy and received a pathological diagnosis were included in the study (M: 244 (69.7%), F: 106 (30.3%)). The average age of the patients was 65.48 +/- 12.32 SD (range: 18-90). Tru-cut biopsy was the preferred method for the majority of the patients (tru-cut biopsy: 244 (69.7%), FNAB: 106 (30.3%)). FNAB procedures utilized the thinnest 20 G needle, while tru-cut biopsies were predominantly performed with 16 G needles to obtain more tissue samples (16 G: 173 (49.4%), 18 G: 86 (24.6%), 20 G: 91 (26%)). A definitive diagnosis could not be established in 38 (10.9%) patients. The majority of diagnosed patients exhibited malignant characteristics (malignant: 268 (76.6%), benign: 44 (12.6%)). Most masses originated from patients with lesions that were inaccessible for diagnosis through bronchoscopy due to their peripheral location. Detailed information on tumor sites, needle entry locations, and pathology results is provided in Table 2.

**Table 2:** Tumor localization and pathological diagnosis

Lesion Features	Number(%)
Pathological Diagnosis	
Non-diagnostic	38(10.9%)
Adenocarcinoma	149 (42.6%)
Squamous Cell Carcinoma (SCC)	64 (18.3%)
Small Cell Lung Carcinoma (SCLC)	21 (6%)
Metastasis	27 (7.7%)
Lymphoma	7 (2%)
Anthracosis	7 (2%)
Infection	37 (10.6%)
Localization	
Right upper	126 (36%)
Right middle	16 (4.6%)
Right lower	66 (18.9%)
Left upper	82 (23.4%)
Left lower	60 (17.1%)

The mean dimensions of the biopsied lesions were 35.98 +/- 17.90 SD (range: 5-105 mm), and the distances to the pleura averaged 13.48 +/- 13.54 SD (range: 0-86 mm). PET-CT results were considered before the biopsy, and efforts were made to extract the biopsy sample from the region with the highest and most homogeneous SUVmax value in the PET-CT. Out of the patients, 240 (68%) underwent a pre-procedural PET-CT scan, while 110 (32%) did not. Among those with PET-CT, the mean SUVmax was 6.65 +/- 6.34 SD (range: 1-39.20).

The majority of procedures utilized a thicker needle, notably 16 G, in tru-cut biopsy, and based on our patient cohort in this article, the complication rate was remarkably low. All complications spontaneously resolved without the need for invasive procedures. Patients experiencing complications were admitted and observed for one day, with all being discharged the following day. Further details on the complications are presented in Table 3.

**Table 3:** Complication types and rates

	Number (%)
Complication	
Yes	80(22.9%)
No	270 (77.1%)
Complication type	
Pneumothorax	57 (16.3%)
Parenchymal hemorrhage	31 (8.9%)
Pneumothorax+Hemorrhage	8 (2%)
Undergone invasive treatment	--
Spontaneous recovery	80(100%)
Factors Associated with Complications	
- Gender (male)	68(85%)
- Distance from pleura	≥22.20 mm

Upon categorizing the pathology results of the biopsied lesions into benign and malignant, a notable prevalence of malignancy was observed in males (56.8%), particularly in Tru-cut biopsies (72.7%) and 16G needle Tru-cut biopsies (47.7%). Malignancy was also higher in cases evaluated by PET-CT before the procedure (59.1%). Furthermore, a statistically significant difference was identified between malignancy, with the SUV max value and the lesion size demonstrating a significance level of p<0.001. However, no statistically significant difference was noted between malignancy and the lateralization of the lesion (left or right lung) or its specific segment within the lung (p: 0.223).

When considering complications, it was observed that 270 (78%) patients did not experience any complications, while 80 (22%) patients had complications. The most significant factor contributing to complications was the distance to the pleura (p<0.001). The average distance to the pleura for lesions in the 80 patients with complications was 22.20 mm, compared to 10.90 mm in those without complications. When comparing FNAB and Trucut biopsy, there was no statistically significant difference in terms of the risk of developing complications (p: 0.734). Similarly, no significant difference was found among different core biopsy needle thicknesses (20 G, 18 G, and 16 G) in terms of complication development (p: 0.638).

In examining other potential variables associated with complications, it was noted that males experienced complications more frequently (75%) (p< 0.001). The needle insertion site varied according to the patient's position, but no statistically significant difference was detected between the needle insertion site (anterior/posterior or lateral decubitus) and complications, nor between complications and the localization of the mass within the lung segment (p: 0.454, p: 0.104, respectively). Additionally, there was no significant difference in the development of complications between lesions evaluated with pre-procedural PET-CT and the pathology results indicating benign or malignant masses (p:0.442, p:0.281, respectively).

No statistically significant relationship was identified between the types of lesions evaluated for biopsy, considering age, size, SUVmax values, known pathology results, and the development of complications. Notably, no statistically significant difference was found between needle thickness and the development of complications (p: 0.478).

The most common complications were pneumothorax and parenchymal hemorrhage, as detailed in Table 2, with pneumothorax (Px) being the most prevalent at 16.3%. When pneumothorax and parenchymal hemorrhage were separately compared, there was no significant difference between FNAB-Trucut or needle thickness. However, the distance from the pleura was identified as the most crucial factor for both types of complications (p:0.002, p <0.001, respectively).

In comparing SUVmax values based on definite pathology results, significant differences were observed between certain types. According to the statistical results, significant differences were found between adenocarcinoma and metastases, adenocarcinoma and infection, and SCC and infection in terms of SUVmax values. Specifically, the highest average SUVmax value was identified in SCCs (8.09), followed by adenocarcinomas (7.71), metastasis (4.96), and the lowest in infectious processes (3.86).

Furthermore, a statistically significant difference was observed between the size of the lesions and the pathological results, specifically between adenocarcinoma and anthracosis, adenocarcinoma and infections, SCC and anthracosis and infection, and SCLC and anthracosis. According to these findings, the average size of the largest lesion was SCLC (44.71 mm), followed by adenocarcinoma (39.44 mm), SCC (39.08 mm), lymphoma (33.93 mm), metastases (32.59 mm), infections (26.46 mm), and the smallest size being anthracosis (19.14 mm).

## Discussion

Today, individual target therapies are preferred over systemic chemotherapy in the treatment of lung cancer. Therefore, the method selected in biopsy procedures is aimed more at determining histological subtypes and performing molecular biological studies rather than diagnostic evaluation.

FNAB can provide a diagnostic diagnosis based on cytological samples; however, tissue samples cannot be taken. Therefore, biomarker analysis and determining cancer type/subtype for clinical studies are not possible with FNAB. Considering this, its value in diagnosis and guiding treatment is lower than that of tru-cut needle biopsy [11]. In a tru-cut needle biopsy, there is a higher chance of obtaining enough tissue samples. Therefore, molecular tests can be performed in addition to pathological diagnosis, making it much better than FNAB in guiding treatment [12]. Sample collection for biomarker studies and biobanking is not possible with FNAB either. Additionally, FNAB is insufficient for specific benign lesions to be diagnosed (e.g., hamartomas) or for a diagnosis of lymphoma [13].

For all these reasons, TCNB is becoming increasingly preferred nowadays. However, when doing a cutting needle biopsy, the aim is to collect the largest possible quantity of tissue (in terms of thickness and length). This sample may provide an indication for up to 70% of patients who can be treated with non-surgical medical treatment. With the progress in biological diagnosis, mutations are being looked for in a greater number of genes; this can only be achieved with larger biopsy samples.

Interventional radiologists doing transthoracic needle biopsy prefer doing biopsies with 18G thickness needles more often. In this present study, however, the majority of transthoracic needle biopsy was done with 16G needles (66%). A small number of studies in the literature with sampling with needles thicker than 18G have been reported [14,15]. However, the needle thickness used for sample collection is of utmost importance as it determines the treatment. For example, when needle thickness is increased from 19G to 18G, tissue sample collection increases by 49%, and when sample length is increased from 10.9 to 12.6, sufficient tissue for molecular testing increases from 85% to 98% [16]. In another study, when the biopsied sample size was increased, the rates of malignancy diagnosis increased from 62% to 76.8% [17].

For specific target therapies in lung lesions, cutting needle biopsy is sufficient to obtain tissue samples and perform molecular biological studies, as in other organs. Additionally, the lesion to be biopsied is not homogeneous but heterogeneous. Attention should also be paid to sampling from the most aggressive and metabolically active part of the lesion. For this, metabolic imaging techniques such as diffusion-weighted imaging (DWI) and PET/CT are used to identify lesions with different activities. As can be seen from these imaging techniques, the lesion has different parts with different activities. Therefore, the most homogeneous metabolically active area should be sampled for the most tissue samples [18,19]. In our study, we took this into consideration in determining biopsy localization and determined that the malignancy rate of lesions with high SUV max values was higher. In addition, our study also showed that the SUV max values were different between the malignant types. The highest SUVmax value was in squamous cell carcinoma (SCC), and the lowest was seen in metastasis.

Considering the complications after lung biopsy, the most common are simple pneumothorax, pneumothorax requiring chest tube insertion, pulmonary hemorrhage, and hemoptysis. In our study, no patient developed pneumothorax requiring chest tube insertion. When the complications and causes were examined, studies presented different results ranging from 9-54%, with pneumothorax, in particular, standing out [20]. In the largest study reported with 15,865 cases, the pneumothorax rate was reported as 15% [21]. The pneumothorax rates seen in our study (16.3%) were not much different. In most studies, the common point is that when the causes of pneumothorax risk are examined, the lesion being deeply situated and of small size (less than 2 cm), the presence of emphysema, and the experience of the practitioner are the most common causes [20,22]. Although it has been suggested that an increase in needle thickness increases the risk of pneumothorax, it could not be fully confirmed. In our study, the most common cause of pneumothorax and parenchymal hemorrhage was the distance from the pleura and in order of frequency.

In our study, the most common cause of complications in pneumothorax and parenchymal hemorrhage was the distance from the pleura, and the complications were pneumothorax and parenchymal hemorrhage, in order of frequency. There was no significant difference in pneumothorax development rates between FNAB (21.7%) and TCNB (23.4%) or between different needle thicknesses in patients who underwent TCNB. It has been reported in the literature that the risk of parenchymal hemorrhage increases with the deep localization of the lesion and the increase in needle thickness [23].

Study limitations

Our study had some limitations. The first was that it was a retrospective and single-center study. Most of the patients were from oncology meetings and the preselected malignancy gave selection bias to our patient diversity. Performing all procedures by one experienced interventional radiologist may have resulted in a significant reduction of complication rates compared to cutting needle biopsy. The absence of a pathologist during the biopsy and keeping the samples in formaldehyde and spread in the presence of a radiologist may have had an effect on the result of FNAB. If samples are taken from the border, the thickness of the needle increases and the risk of hemorrhage will decrease.

As a result, in the most common type of cancer, lung cancer, which has a high medical treatment rate, individual target therapies are rapidly replacing systemic chemotherapy to prolong survival. Molecular studies that could be used for the application of these more effective treatments require the largest pieces possible from the appropriate location, but this is only possible with TCNB. After carefully evaluating pre-biopsy CT and PET-CT, it is possible to safely obtain sufficient tissue samples from most lung lesions with 16G needles.

Main points

The critical initial step in diagnosing lung cancers and determining targeted therapy through molecular biological studies is the size of the biopsy sample. To ensure the efficacy of this diagnostic process, it is imperative to utilize the thickest needle available and extract the longest sample during lung biopsies. Additionally, pre-imaging techniques should be employed to identify the most suitable biopsy localization, emphasizing the importance of precision in the diagnostic procedure.

## Conclusions

The size of the biopsy sample taken for the purpose of determining targeted therapy in molecular biological studies for the diagnosis of lung cancers is the first and most crucial step. Therefore, the thickest needle and the longest sample should be used for lung biopsies, and the most suitable biopsy localization should be determined through pre-imaging techniques.
